# Os Joelhos! Os Joelhos! Protective Embodiment and Occasional Injury in *Capoeira*

**DOI:** 10.3389/fsoc.2020.584300

**Published:** 2021-01-14

**Authors:** Sara Delamont, Tiago Ribeiro Duarte, Issie Lloyd, Neil Stephens

**Affiliations:** ^1^School of Social Sciences, Cardiff University, Cardiff, United Kingdom; ^2^Department of Sociology, University of Brasilia, Brasilia, Brazil; ^3^Independent Dance Instructor, Bristol, United Kingdom; ^4^Social and Political Sciences, Brunel University London, Uxbridge, United Kingdom

**Keywords:** embodiment, ethnography, capoeira, illness narratives, injury narratives, martial arts

## Abstract

*Capoeira*, the African-Brazilian dance and martial art has enthusiastic devotees in Britain. Most practitioners are acutely aware of their *capoeira* embodiment, and have strategies to protect themselves from injury, and ways to seek treatment for any injuries they get. Drawing on data from a long-term ethnography and a set of 32 open-ended interviews with advanced students, the paper explores student strategies to prevent *capoeira* injuries, and their discoveries of effective remedies to recover from them, before it presents an analysis of their injury narratives using Frank's three-fold typology of illness narratives. The *capoeira* study therefore adds to the research on sports and dance injuries, and to the intellectual debates on the nature of narrative in research on illness and injury as well as exploring one aspect of the culture of *capoeira* students in the UK.

My knee stopped me for a few months, my back stopped me for 6 months, my shoulder stopped me for 6 months, and a car accident stopped me for 7 months.

(Penstemon, female, fifth belt in 2016)

## Introduction

*Joelho* is the Portuguese word for the knee. *Capoeira*, the African-Brazilian dance-fight-game, uses a predominantly Brazilian Portuguese vocabulary, including songs in Portuguese so its serious students learn the Brazilian Portuguese names for its moves and for body parts like the knee. The knees are one of the body parts that practitioners of the African-Brazilian martial art capoeira report having trouble with. Both teachers and students are familiar with knees getting injured, or throughout their regular training realizing that they are gradually more insidiously becoming painful. There is even a traditional capoeira song that includes the refrain:

Jogue comigo com muito cuidado

Com muito que meu joelho tá quebrado

“Play gently with me, my knee is broken.”

Any capoeira teacher or learner would sympathize with that sentiment: painful knees are a common problem. Capoeira, though often called a martial art, is also a dance and a game, and its enthusiasts (*capoeiristas*) are players rather than fighters. In Brazilian Portuguese the verb used is *jogar*—to play—not the verb for fighting. This paper draws on a body of interview material collected from students about the experience of injuries, strategies to avoid them and ways to deal with any injuries that do occur. These 32 interviews are set in the context of a 16 years long ethnographic study of contemporary capoeira as it is taught in two British cities—Cardiff and Bristol—by *Mestre* Claudio Campos, a Brazilian master. All the respondents were experienced capoeira learners in the two British cities.

The woman we have called Penstemon, whose interview is quoted at the head of the paper, is one of the most persistent capoeira students in the group we study. Few students remain so keen on doing capoeira if they have suffered that many injuries. At the time of her interview in 2016 it was 10 years since she began training, but she had “lost” 2 years of her involvement because of these four major injuries. At the time of the interview she was waiting for an operation on her thumb, which would necessitate another break. That was successful and she is still training at the time of writing. Our analysis draws on literature about illness narratives (see Thomas, 2010) and research on how injuries are reported by enthusiasts for other sports such as rugby (Smith and Sparkes, 2005) and martial arts practitioners (Smith, 2008).

Casual observers could think that capoeira is a dangerous activity, and that its practitioners would get injured. In Brazil capoeira games in the streets between men can be aggressive, but outside Brazil capoeira is taught as a non-contact activity. Men, women and children train and play together, escapes are as important as the attacks and the acrobatic flourishes are taught, practiced, and drilled extensively. Unlike many South East Asian martial arts kicks are not routinely blocked, but dodged, and punches or blows with the arms and elbows are rarely used at all. Attacks with the head and knees do exist, which, like kicks, can be blocked, but are equally often evaded. Protective clothing, like helmets, shin guards, or gloves, is not worn, nor are gum shields, and it is not mandatory to remove or put plaster over jewelery as it is in Savate classes (Southwood and Delamont, 2018). Learners who wear glasses can train wearing them, if they choose to do so.

Capoeira training involves learning and drilling movements individually in lines, paired practice of sequences, and games in the *roda*, a circle of *capoeiristas* and a group of musicians (*bateria*) within which two people interact using the attacks and escapes they have learnt (Delamont et al., 2017). Students *are* exhorted to train and play with one arm positioned to protect their face and head, as a precaution. In Claudio Campos's classes the instructions “Protect yourself” and “Protect! Protect!” are frequently heard, along with advice to escape as low to the ground as possible. Students are routinely told that capoeira can be dangerous. Games in the UK with strangers, who may have been trained in a more aggressive style, and all encounters in Brazil are likely to be fiercer and could involve more contact and more “real” take downs. Such contrastive rhetoric works to stress how “safe” capoeira is *here*, as long as you concentrate, use dodges and protect yourself but outside the home club, *there*, it could be very dangerous. The injuries capoeira students and teachers get doing capoeira are mainly sprains, pulled muscles and damaged ligaments and tendons, usually reported as due to falls in training or games or failing to take sufficient care when attempting moves. During our fieldwork injuries due to capoeira encounters have been very rare. At the time of writing (March 2020) Delamont has observed Claudio Campos teach 681 times, and watched 464 other classes, and has never seen an injury worse than a black eye. In the Bristol class 15 years ago a man got his cheek bone fractured by a spinning kick from another student who was training next to him and the fact that this is remembered in the oral culture shows how rare injuries are.

The authors are three capoeira practitioners (Duarte, Stephens and Lloyd) one of whom is Brazilian, and a non-playing ethnographer (Delamont). All three capoeiristas have had injuries themselves. Capoeira does produce a toll on bodies—the teachers (professionals), especially those over 50, often report problems with their knees and hips. Mestre Gato, a pioneer of capoeira in the UK, has had both his hips replaced. Teachers are not, of course, immune from getting injuries from non-capoeira activities. They have bicycle and car accidents, and get hurt doing other activities. As a young man, in Brazil, Mestre Claudio Campos got several injuries when doing Brazilian jiu-jitsu and so stopped doing it to focus on capoeira. In the UK he injured his knee playing soccer and had to have surgery.

The paper begins with a brief account of *capoeira*, and a short methods section followed by an overview of our results. The heart of the paper is the debate among social scientists about illness narratives, the analysis of the data using the ideas of Frank (2012), and the insights gained into the habitus of diasporic capoeira. Frank (2012) suggested that illness narratives could fruitfully be classified into one of three types, quest narratives, chaos narratives and restitution narratives. This classification is useful for organizing the injury narratives we have collected from capoeiristas in the UK. Many sociologists of the body draw on Bourdieu's (1977) concept of habitus as we have done elsewhere (Delamont et al., 2017). He argued that research on how new bodily habituses were acquired was essential to understand embodiment and performativity. We argue here that the analysis of injury narratives is a way to gain insight into the embodied habitus of capoeira.

## Context: Capoeira Outside Brazil

Across the USA and Canada, in Europe and in Australia and New Zealand, thousands of people are eager to learn new and “exotic” forms of embodiment and performativity from other cultures. They dedicate time, energy, and money to acquiring skills and knowledge about “other” bodily traditions, such as Argentinian Tango, Chinese Kung Fu, Spanish Flamenco, Japanese Aikido, Scottish Highland dance, or Brazilian Capoeira. Men and women who decide to learn an “exotic” dance (such as Salsa) or a martial art (such as Muay Thai) are embarking on a journey into a new culture, as well as a project to make new friends and learn new bodily skills. Their teachers are either expatriates from the homeland of the exotic dance or martial art or “native” British people who have fallen in love with the dance or martial art in their youth, become experts in it, traveled to study it in its homeland and other centers of excellence to gain authentic experience and “qualified” formally or informally, as teachers. This paper draws on a larger ethnographic investigation (Delamont et al., 2017) of contemporary capoeira classes in the UK focused partially on the re-enculturation processes engaged in by novices acquiring new bodily habituses.

Capoeira's origins are undocumented and disputed (Assunçâo, 2005). Elements of capoeira, such as the musical instruments, the call and response singing, and the circle in which games are played (the *roda*), are certainly African in origin. The specific activity of capoeira is African Brazilian, developed by the slaves on the plantations of Brazil, or in the communities set up by escaped slaves, or in cities such as Salvador de Bahia. It was a male street pastime in the cities after the slaves were “liberated” in 1888, and was made illegal. In the 1930s it was legalized and recognized as a good way to get the armed forces and school children fit via a “Brazilian” (and not a colonial Portuguese) activity. In that era the so called “new state” led by Vargas (1937–1954), two varieties developed: *regional* which incorporated some aspects of South East Asian martial arts and *angola* which self-consciously aims to be true to the authentic roots of capoeira. *Regional* developed, in the period after 1970, into a variety called contemporary (*contemporânea*), which is today widespread across the globe. The classes we study are capoeira contemporânea. During the rule of the military junta in Brazil (1964–1985) many Brazilians went into exile, and took capoeira with them to North America, Europe and Australasia. There has been capoeira in the UK since the 1970s, with regular classes in Bristol since 2001, and in Cardiff since 2003. Many cities in the UK have, in 2020, at least one capoeira teacher running a group. In UK capoeira most of the teachers are Brazilians, but there are people of other origins who have chosen to dedicate themselves to the Brazilian dance-fight-game. Our research has been conducted primarily with Mestre Claudio Campos, a Brazilian who has taught in the UK since 2003.

Nicknames (*apelidos*) given by the instructor, are common in diasporic capoeira. Delamont and Stephens have “real” nicknames but routinely publish with the Portuguese pseudonyms *Bruxa* (Witch) and *Trovão* (Thunder). Pseudonymous nicknames are used for all informants in this paper. Men have nicknames from Kipling's *The Jungle Book* and women the names of flowers and trees. This gender difference in our pseudonyms reflects the way in which teachers allocate nicknames: men get funny or heroic names, women pretty ones (see Owen and de Martini Ugolotti, 2019). These are not sources of nicknames used by any UK capoeira teacher we have studied. “Real” nicknames are generally in Brazilian Portuguese, such as *Sorvete* (Ice Cream) or *Grego* (Greek). Teachers mentioned here have pseudonyms from Greek or Roman mythology and history such as Claudio and Seneca.

Most of the ethnographic research has been conducted on capoeira angola, in its heartland in Salvador de Bahia, Brazil (Lewis, 1992; Downey, 2005; Willson, 2010) or overseas (Griffith, 2016; Varela, 2017a,b). In capoeira, as well as being drilled in the “fighting” techniques, all students are expected to learn several dance forms and perform them in public demonstrations of capoeira related activities and at social events. The basic techniques of capoeira are attacks (such as kicks), escapes, and acrobatic “flourishes”. Headstands, cartwheels and handstands are used routinely, while more elaborate acrobatics, such as a high back somersault are rarer, used only by the most physically skilled people, and are mainly used in displays to “amaze” an audience. Classes, which usually last 90 min or 2 h, consist of drill and practice in lines, or in pairs, of individual moves or short sequences, and often end with “a *roda*”: when pairs have an opportunity to play free games before their peers. Capoeira has an extensive virtual presence, with films, DVDs, websites and music downloads enabling those far from Brazil to establish themselves in a global network (Delamont et al., 2017).

## Research Methods

The ethnography is about capoeira as it is taught and learnt in English speaking countries (the UK, Canada, New Zealand) far from Brazil (Stephens and Delamont, 2006a), but the data on injuries were collected by interviews. The ethnographic methods are described in detail in (2017 Appendix One 178–191). The material used in this paper, on students' preventive strategies, the treatments sought when they were injured, and their accounts of injuries cannot be observable directly as they do not occur in capoeira classes. So the main data analyzed here are 35 injury narratives drawn from the 32 interviews. The informants were learning capoeira as a hobby in two UK cities and are mostly British. The interviews were conducted in two phases in 2013–2014 by Duarte a Brazilian sociologist who does capoeira, and by Lloyd, a Welsh dance teacher and experienced capoeirista. Duarte had not done capoeira in Brazil, but began to learn it in Cardiff during his period as a Ph.D. student. He has suffered recurrent back injuries. Students were eligible to be interviewed if they had been learning capoeira regularly, and therefore seriously, for over 2 years and had been awarded at least the second belt (*corda*) in the group's hierarchy explained below. Many people start capoeira, and may get the initial blue belt, but these interviews were designed to capture the experiences and narratives of serious, persistent students. The Duarte interviews covered a range of capoeira topics including injuries. Lloyd's interviews were focused on injuries specifically to provide data for this paper, and we obtained 35 injury narratives from 32 interviews. In the 2016 wave there were 12 women respondents and eight men, in the earlier phase there were seven women and five men who discussed their preventative strategies, injuries and recovery histories with Duarte, and so only their responses are included here (see Table 1). The longest any respondent had been training when they were interviewed was 18 years, the shortest three, the average six but some had trained continuously while others, like Penstemon, had had gaps.

**Table 1:** The two sets of interviews.

	**Average Years of training**	**Range**	**Range of cordas**	**Total**
**Injury enquiry 2016**				
Women	6	3–12	1–5	12
Men	6	3–18	2–5	8
**General interviews 2013–2014**				
Women	5	2–8	1–4	7
Men	7	3–10	2–4	5

Contemporary capoeira groups give *cordas* to students as they progress, and we have labeled all quotes from respondents with their sex, number of years in training and belt level in 2014 or 2016 (the date of their interview). Cordas are awarded at intervals that increase noticeably as the level of skill necessary rises. So the most experienced current Cardiff student Jagai got three cordas in 3 years (2003–2006), was subsequently raised to the fourth in 2010 and to the fifth in 2016. His, typical, gap between third and fourth belts was 4 years, and the next gap was 6 years. Most respondents were attached to Mestre Claudio Campos's capoeira group, but a minority regarded themselves as members of another group, and were training with Claudio because they were living in Bristol or Cardiff.

The interviews for the injury enquiry were semi-structured. Delamont and Stephens devised a topic list that addressed the central issues we wanted to explore. The interviews were recorded and transcribed by Duarte and Lloyd and if originally in Portuguese, translated into English, and then all of them were analyzed by Delamont. We asked about injuries (such as a fractured cheek bone or damaged knee ligaments) that interrupted training, whether due to capoeira or other activities such as snowboarding or soccer. Both of the authors who devised the questions had experienced injuries that interrupted their capoeira training. Stephens had done karate from childhood and has a Second Dan Black Belt in Shotokan karate. He began capoeira in Cardiff as a postgraduate student, and trained for 6 years, gaining the third belt, until recurrent injuries led him to cease training. The respondents knew the interviewers as fellow capoeira students, and in some cases were friends. So these were interviews between people of a similar age and lifestyle, discussing an activity they were both invested in. They were paid £20 for doing the interview and put into a raffle with a chance to win one of four prizes each of £500. There were two prizes for each of Meste Claudio Campos's clubs in 2016. The money was specifically intended to enable the winners in 2016 to go to Brazil for the ceremony at which their teacher Mestre Claudio Campos was being promoted to the highest teaching grade, that of master (*mestre*). For the intended respondents this was an incentive, because Mestre Claudio Campos's long term students wanted to see him rewarded at his elevation.

There is a caveat about the data: we expected more volunteers and many more injury narratives than we actually obtained from the sixty or so experienced learners in the two cities. This absence can be related both to a pattern in the recruitment to the study and to decisions by capoeiristas about continued involvement. This was a feature of the research design, which should have been foreseen, and that, in retrospect, has made the data less valuable than we anticipated. We expected the volunteers to be currently engaged in capoeira with experience of past injuries. In the event we had underestimated how many people who had been injured did not volunteer to be interviewed for the project. Some of those who might have been particularly useful informants because of their serious or recent injuries had stopped training permanently *because of* injuries and no longer self-identified as capoeiristas. They were not motivated to come forward to be interviewed even if they heard about the project. Others with an injury that was temporarily interrupting training were unlikely to want to go to Brazil for a capoeira festival if too impaired to play any capoeira there themselves. Additionally, anyone who could not go to Brazil due to work, study, or family commitments was unlikely to volunteer to be interviewed because it would reduce their friends' chances of winning one of the prizes. These problems meant that we had a set of respondents who were keen to go into the draws so they might win the cost of the airfare, were not currently injured and had not “left” capoeira through injury.

## The Injured Respondents

The majority of the people interviewed had experienced injuries of some kind that had interrupted their capoeira training. For many of the informants, capoeira was an important part of their recent and on-going journey through life itself. At least half of those injuries had occurred outside capoeira: at work, doing other sports and exercise, or in bicycle or car accidents. The capoeira injuries were mostly done when trying an acrobatic move, or were inflicted during games when an attack went wrong. Raksha's interview is used to illustrate a typical story because he is articulate about his experience of an injury and his subsequent responses to it.

The worst injury that I had did actually completely stop me [doing capoeira]… It was a few years ago now, but it was a good few weeks. It was actually down in the gymnasium gym. I was trying to learn how to do front flips. (Raksha, Male, 9 years training, 4th belt in 2016)

He was practicing on what he described as the wrong type of surface

It's less springy and there's less soft stuff to land on, and as I landed I was still in a *cocorinha* [squatting] position because I hadn't managed to get enough height and all of the force went through the side of one of my legs. I would swear that I heard something snap but it was probably just my imagination.

The consequence was

I just couldn't walk properly for a few days, and then it took a long time then to actually do anything.

Raksha had been training for 9 years with gaps due to moving cities, and employment issues. Raksha loves capoeira, particularly the acrobatics, and also does gymnastics and “a bit of circus”. He did not seek any medical diagnosis or treatment and missed training “for up to about a month”. He has not, he says, suffered any “serious” injuries although many readers might classify this as “serious”. Apart from this leg injury Raksha mentions some short gaps due to more minor injuries from capoeira, and gymnastics, and he has a recurrent “slight muscle pull, strain” in his neck and shoulder. He explains

I think it's the place where I hold a lot of tension and if I'm not warmed up properly and not aware enough of my body at the time then I have an inclination to overdo it and—err—compensate.

He mimes lowering himself from a handstand into a headstand, a move his interviewer, Lloyd, can do herself.

Yeah, handstand to headstand and yeah losing a bit of my strength to go down too fast and obviously just cricking my neck. So I ‘ve done that a few times.

Lloyd asked what Raksha does about that recurrent injury, and whether he sought help.

No, no. I would put hot and then cold on it, heat pack and then an ice pack and just keep using creams and stuff.

Rashka also had an injury from working as a house painter, when he got a back seizure. However, compared to Penstemon, with whom we opened the paper, Raksha has had little disruption to his capoeira experience. He was philosophical about injuries:

If I'd have had my knees closer together it might not have happened. If I had jumped higher it might not have happened. You do these things knowing full well….I've had closer calls with things like the flying trapeze at circus school. In Kung Fu I dislocated two or three of my fingers.

The majority of the informants had injury histories between Penstemon's three soft tissue problems and her car accident, and Raksha's leg damage and his recurrent neck pain.

As well as routine classes, which happen four or five times a week, capoeira in the UK has special events—festivals—to which lots of teachers and students come. The following extracts are from the ethnographic fieldnotes taken during a weekend in the Autumn of 2017 when over 100 people congregated in central London. We have drawn on the fieldnotes from the 3 days to illustrate injuries, public prevention strategies, one therapeutic activity, and the passion for capoeira that characterizes the lives of our 32 informants and their coevals. The weekend had been organized by Mestre Joãozinho of *Mar Azul* to honor two African-Brazilian veteran *mestres*: Mestre João Grande and Mestre Moa do Katendê. João Grande was 84 at the time, and Moa do Katendê not much younger, and it was thought that it might be British capoeiristas last chance to see, and learn from, them. Tragically Mestre Moa do Katendê was murdered in a bar the following year after an argument about the presidential election, so that prediction came true. The event, which cost £85 for one evening and two full days (including a celebratory Tshirt), was attended by Delamont, Lloyd as a full participant, and Mestre Claudio Campos as one of the many UK based teachers.

On the Friday evening the two Brazilian masters attended two different events. They went to Mestre Poncianinho's regular Friday night class in Fulham from 6.30 to 7.30, and then attended Mestre Joãozinho regular open roda in West Kensington. This happens every week and is “just” capoeira games. On Saturday and Sunday the two mestres taught huge classes in the sports hall of a London University. Delamont and Lloyd spent the Friday evening at the open roda, but the fieldnotes also mention events at Mestre Poncianinho's class. This was possible because Delamont has often attended Mestre Poncianinho's Friday class, knows many of the students, and was shown photographs of it by regular class members on the Saturday. There were about 50 people at each location on the Friday, and over 100 on each day over the weekend. The fieldnotes for this event run to 47 A4 pages, but we have extracted material related to the themes of this paper. Alongside the “celebrity” “star-studded” aspects of this event, there were multiple mundane encounters that included observation and accounts of injury prevention, of past and present injuries, and of therapeutic encounters.

The Friday night event in Kensington “began” at 5.25 when 10 people started to play capoeira music in an impromptu *bateria*. From 5.25 until 6.05 people stretched and played warm up games with friends, queued to pay and collect their Tshirt, greeted friends warmly and strapped up their ankles, knees and wrists as a precaution. At 6.05 Mestre Joãozinho explained the timetable for the evening and took Mestre João Grande and Mestre Moa do Katendê away to Fulham. One roda formed near the *bateria* and there were 18 players in it by 6.15. People arrived in a steady trickle all evening, paid, warmed up and joined in either as musicians or players. At 6.15 a teacher divided the first roda into two, and at 6.30 a third one was created. The aim of this is to keep rodas small so everyone gets a chance to play games. At 7.07 there were 10 musicians, eight people in the original room, 10 in the second, and six in the third. One woman, who had arrived with her arm in a sling, told her friends she would not play, but at 7.20 she took off the sling and played a careful game with a heavily pregnant fellow student. No one found that odd, because serious capoeira students often train throughout their pregnancies, and injured people often decide to play a careful game.

On the Saturday people began to arrive from 10.30 although the hall was not free till 11.00. Once the large sports hall was free and people could go into it, the organizers set up a *bateria*, a food table for the teachers, hung banners, and those teachers who had brought things to sell (such as DVDs and instruments) laid them out on tables. Individuals warmed up, put on precautionary strapping, played gentle games with friends, and caught up with people. Mestre Joãozinho “opened” the event with a formal welcome to Mestre João Grande and Mestre Moa do Katendê, and then to all the teachers present, starting with the mestres and moving on down through the lower grades to the instructors. All the mestres but one, a Scandinavian, were men. The proportion of women in the lower grades like “Professor” was higher. Mestre João Grande began to teach at 11.40 with a set of exercises that were capoeira but were also stretches, done individually and then in pairs. João Grande is now too old to do a lot of the moves he wanted the students to undertake, so he has two African-Brazilian women who demonstrate each move to the class for him. This is normal if a master is old and frail, or injured. All the capoeira teachers present either played music or took the classes alongside the students. The first pair in the class to try the required exercise are themselves teachers. Teaching stopped for a lunch break at 1.45 and at 2.30 a music class took place, with 35 *berimbaus* being played all at once. Unusually Delamont was not the only capoeira researcher present, as Assunçâo (2005) took classes but also conducted interviews with teachers present throughout the event.

Sunday's event was due to start at 10.30 but at that point only 12 students were present including three who set up a set of screens and two massage tables at the far end of the hall opposite to the *bateria* which were occupied all day by people having massages which they paid for. This is not unusual at special events. Many capoeira groups include people who are trained to do massage, and it is common for them to set up a portable table and work, so that other students can be massaged *in situ*.

The formal proceedings began at 10.53 with welcomes, and then Mestre Moa do Katendê led a dance class, a common event at the beginning of the second full day of festivals which warms everyone up, stretches their bodies, emphasizes the African and African-Brazilian origins of capoeira and the need to move with the music. The massages and the dance class are the most public aspects of general preventive strategies recorded that weekend. On the second day of an event when many students are tired and aching from the intensive work on day one, to begin the second day with a dance class and a long and careful session of stretching, before any formal capoeira teaching takes place.

The capoeira classes begin at 11.24 −60 people are training, plus a bateria, and the people having massages. At one point a Brazilian mestre who teaches in the north of England goes to get massaged telling Delamont that because of the long drive to London that morning he needed to get his back “sorted” before he did any capoeira. Prevention strategies are employed by teachers as well as students.

At 12.13 Mestre Moa do Katendê called the class to a roda, which is mainly teachers, and many students take photographs of the games. A short break is then followed by Mestre João Grande teaching again. One of the paired sequences the mestre gets his advanced helpers to demonstrate involves one of the pair doing the splits as their opening gambit, and then when they change roles their training partner starts the sequence from the splits. A high proportion of the students present cannot, or choose not to, do the splits. Avoidance of a move that risks injury is normal.

A young woman who got injured on the Saturday (she hurt her leg training) is here today and she has a massage.

At 2.34 there was a lunch break and classes resumed at 2.53, with three rodas formed. Games are played, but Mestre João Grande occasionally stops them to make a pedagogical point, improve the singing, or get his helpers to demonstrate a move he has seen done badly. At 4.06 the bateria was all women. A small man wearing glasses was in a game with a tall blonde man from one of the groups based in mainland Europe, whose style was very aggressive, and who ignored all the small man's signals to calm the game down. When Delamont spoke to the small man after the game he said he had feared he might get an injury, but, he added “Perhaps I've insulted his mother”, and “there are lots of styles.”

During the course of the two and a half days four students pulled out of a class or a roda with an injury of some kind, and in three of those cases ice packs were fetched from Reception for them. At the beginning of each session many people strapped their knees, or ankles, or both as a routine preparation for the exercises. Delamont was told of three people who had not come because they had injuries, and eight people talked to her about their state of health, including Penstemon (the flower), the student whose “history” began this paper, who had had her thumb surgery and was training again very happily. There were no “serious” injuries: no one left the event, or went to hospital. Our sociological interest in this account, and the interviews, lies in the students' prevention strategies, to which we now turn.

## Illness and Injury Narratives

In this section we relate our injury data to the literature on illness narratives and the appropriate social science strategies for collecting, analyzing and writing about them. Injury narratives are a feature of two different areas of social science research: dance and sports studies and the sociology of health and illness. Injury narratives can be seen as a variety of illness narratives, a topic of controversy in the sociology of health and illness, and the wider discussions of interviews more generally (Atkinson, 1997, 2009; Atkinson and Silverman, 1997; Silverman, 2017). Frank (1995, 2010, 2012) argued in a series of influential papers and books that social scientists should collect, and showcase, the narratives of patients to rebalance the social science study of health and illness, which he saw as too focused on the standpoint of the doctor. Frank, himself a cancer survivor, was particularly important in establishing narrative based studies of patients with chronic and life-threatening conditions such as epilepsy and cancers. Frank's (1990, 1991) earlier work on the disciplined body has been used to explore the embodiment of capoeira masters and learners drawing on symbolic interactionist ideas (Stephens and Delamont, 2006b) but his analyses of illness narratives have not been applied to capoeira injuries and their consequences before. Before we draw on Frank's work on such narratives we have set it against a dispute among interactionist social scientists about the appropriate use of them.

Atkinson (1997) argued controversially that collecting and showcasing patients' stories was not, in itself, a defensible or useful *social science* activity. He pointed out that the task of the anthropologist or sociologist was to collect the data with social scientific research questions in mind, to analyse the narratives, and to write theoretically informed analyses of them. An absence of social scientific analysis, Atkinson argued, was an abrogation of social scientific responsibility, because the job of a social scientist is to analyse data, and to generalize and theorize from them. Merely celebrating the voice of patient is not, Atkinson argued, social science. Atkinson and Silverman (1997) made a more general argument about interview data in contemporary western societies, making the same call for rigorous analysis repeated recently by Silverman (2017). Frank (2010, 2012) and Bochner (2001) accused Atkinson of both lacking in human feeling and empathy, and being a naive empiricist or an apologist for, or an ally of, western capitalist medicine. Thomas (2010) is an overview of that dispute with replies by Atkinson (2010), Frank (2010), and Bochner (2010). Frank (2012) is a further reiteration of his view. In contrast to the sociology of health and illness injury narrative research in dance and sport does not have authors arguing that celebrating the voice of the “wounded” is a “sufficient” task for social scientists. The studies of injuries suffered by sportsmen (Smith and Sparkes, 2005) and ballet dancers (Turner and Wainwright, 2003) are analytic not just celebratory. Sparkes and Smith (2012) for example *analyse* their data on spinal cord injuries among male rugby union players using Frank's typology.

Frank (1995, 2012) reported that there were three main types of illness narrative: restitution, chaos and quest. In the first type, the patient tells how they were ill, were diagnosed, treated, and were able to resume their lives in an acceptable way. In these stories medical staff are represented as largely positive figures. In chaos narratives, the protagonist finds his or her life collapsing due to illness and many other issues. Everything that is tried makes things worse, and the result is chaos. Medical professionals are among many actors in these narratives who “fail” the narrator and are portrayed negatively. Smith and Sparkes (2005) found chaos narratives in their data on male rugby players who had suffered life changing spinal cord injuries. Quest stories are more like traditional folk and fairy tales, where the protagonist seeks something (Atkinson, 1992, 1996; Delamont, 2009) such as Gerda setting out to rescue Kay from the Snow Queen, or the dwarves in *The Hobbit* trying to regain the Arkenstone and other treasure held by the dragon Smaug. In such traditional quest stories the seeker is transformed by the experience, and often comes to accept that it was their destiny to be the seeker. In illness narratives the quest to regain good health is transformative for the seeker and something equivalent to “treasure” is discovered or gained by the main protagonist. In some of the injury narratives we collected, the capoeirista has learnt how to train and play regularly while preventing further injuries, and claims to have achieved life-changing knowledge of how to look after their physical and mental welfare a successful quest. We decided to explore the capoeira injury narratives to see if these three types existed in that body of data.

We hypothesized that those in our sample were most likely to tell us restitution narratives or quest narratives, because capoeiristas with chaos stories had probably left capoeira so we would not find chaos narratives in our data set. There were twenty distinct stories about health-related events that had interrupted the capoeira training of women, three of which were about pregnancies. In the interviews with men there were fifteen narratives about injuries that had prevented capoeira training. For both sexes the majority of the injuries had been suffered in capoeira related activities such as games in *rodas*, and the minority during other activities either sporting (snowboarding, gymnastics) or in traffic (especially while cycling). Excluding the pregnancies we had 35 injury narratives available for analysis. We found one story we could perhaps classify as a chaos narrative, although the same man had two other restitution stories as well. Only two stories could be classified as quest narratives. All the rest were classic restitution stories, of the kind we were expecting to hear from these informants one of which we analyse below.

We have focused on one restitution narrative here, drawn from an interview Lloyd did with Fuchsia. She began capoeira with Mestre Claudio Campos in 2010 and was on her second belt in 2016, having lived in another city for a year and trained as a visitor with a different group. Fuschia has, since her interview, been raised to the third *corda*. There were four injuries she chose to talk about, two capoeira and one skiing incident and a traffic accident. Fuchsia had had two leg injuries, one in her thigh and a second in her calf, and had stopped training for 2 months with the first injury, and three after the calf injury. Her account began:

A torn muscle in my thigh, that was the first one actually that was playing capoeira. Because I was really tired and exhausted. I pushed myself too hard in the class.

For that injury Fuchsia first saw a chiropractor because she thought she had a back injury, but discovered: “It was really the muscle that was torn!” So she went to a sports therapist. Fuchsia blamed herself for the injury:

It was a bad time mentally as well, I was trying to quit a job that was super stressful and trying to give myself something that I like then pushing myself too much so it was definitely my fault.

The chiropractor was “so expensive” but Fuchsia was pleased she had consulted him because he was honest, because he diagnosed the problem as muscular and recommended a good sports therapist.

The second injury began with a torn calf muscle but also led to knee pain. Fuchsia was again clear that it was her fault.

It was in a festival but I think it was my fault because I was feeling a bit under the weather. I was a bit ill and pushed because it was a festival so yeah, I know it was me, I think I had a cold.

This injury was to her other leg. She again saw the sports therapist who got her to do exercises with an exercise belt and then gave her painful massages that were beneficial. So the second story was also a restitution one. Fuchsia then had her third problem, a knee injury while skiing, which meant “the knee was unstable” so

I went back again to the sports massage guy.

This time she was away from capoeira for at least 6 months. Fuchsia produced her longest account of that injury. Initially she did not have any treatment because she thought:

I really need to heal this [but] I cannot afford any more [treatment]

However, after some months she went back to the sports massage therapist

I was worried if I'd ever be able to train anything again

The therapist said

You need to strengthen the muscles and I said “But I don't want big muscles” and he said “No, no, you have a normal diet so don't worry but do the exercise because it's the only way to keep the knees healthy.”

The exercises did not leave Fuchsia pain-free

So after he did everything that he could he said “well take an MRI scan with the NHS” and I said I really wanted to do this otherwise I'd have to pay like £200 for it or something. So I went and they didn't see anything and in between I was a bit scared and I was thinking that I absolutely don't want an operation. So I tried acupuncture and in one session my pain I had when I used to bend: there was a sharp pain that was there and holding one back all the time. In the first session the pain was a lot less! Incredible! Incredible! So whatever happened I did 6 or maybe 12 sessions of acupuncture and that's been the best.

Lloyd asked if Fuchsia would have acupuncture again.

It was really good the acupuncture—I had never tried it before and was not very keen on having anything stuck in my body, but really on my first session I was quite surprised—it worked!

The third part of the overall narrative covered a year of Fuchsia's life, and she then suffered whiplash in a car accident and went to a different chiropractor. Since then Fuchsia has been careful to warm up thoroughly, and if she feels any pain in class stops training and goes to the side to stretch. All her recoveries have been

Totally worth it [because] capoeira is good for the soul…it is quite incredible—it's a display of energy

This interview displays the characteristics of a restitution narrative. It has a happy ending, and both the orthodox and alternative practitioners are positively portrayed. Fuchsia reports that they all tried hard to diagnose her injuries and offer useful strategies to cure them. The diagnostic and remedial processes produced positive outcomes and eventually she can train regularly again.

Fuschsia's restitution narrative contains the elements of the majority of the respondents' accounts of their prevention strategies, and their recovery strategies.

### Preventing Injury

Many of our informants prefaced their responses about how they prevented injuries with statements about how “lucky” they had been to have avoided body damage. There are six main prevention strategies reported in the interviews, readily observed in every class, and commonly discussed. They are: strapping and bandaging, intensive stretching before and during class, yoga or pilates, avoiding moves that are potentially injurious, “listening to my body,” and relying on *axé* which is a capoeira term we explain below. Strapping and bandaging are widely used. At the beginning of every class it is routine to see people pulling on commercially made ankle, knee and elbow supports, or strapping those joints with bandages. Rashka rehabilitated his ankle by wearing an ankle support for some weeks, and thinks that wearing trainers, rather than going barefoot, also helped his recovery.

Intensive stretching is normal. Most classes start with a warm up, especially in colder weather or chilly halls.

The prevention strategies use at home outside formal capoeira classes are not a visible part of the ethnography. The careful attention to stretching and warming up that are a feature of Claudio's classes make recurrent appearances of our fieldwork, and as Claudio has aged (he is now over 40) he pays more attention to ensuring that he and his students are ready to learn capoeira safely. Such practice is evident in the following fieldnotes, made on August 1st 2018 in Cardiff. There were 18 students present when the class began, including a student (Marut) who had been in Mestre Campos' class when he first came to the UK in 2003, who was back in Cardiff from his home in Scotland for a family visit. The experience level was wide. Marut and Jagai had been training for 15 years, while three students were complete novices at their first ever class.

Before any general activity people strapped their knees, wrists or ankles ready for the class. At 8.09 M. Claudio set the class to run clockwise round the hall, forwards, backwards, forwards, sideways facing in, forwards, sideways facing out, forwards touching the floor with alternate hands, forwards lifting the knees up, forwards lifting the heels up, and then backwards again. It was a warm summer evening, and the hall was hot even with the fire doors open, so no one needed to do violent exercise to get warm. At 8.12 Claudio spread the students out in lines facing him and began to lead and demonstrate a set of stretches of each area of the body.

At 8.13 three more students arrived and joined the lines. The stretches include the dive (*mergulho*) in which the person lies on the floor on their front with the upper body and arms raised off the floor. Then Claudio demonstrates raising the legs as well as the upper body and holding their feet with their hands, the students begin rocking on their stomachs.

Other stretches based on standing and balancing alternate with several bursts of 10 star jumps counted in Portuguese, and press ups, leg pulls, and stomach curls (50 of each) plus various ways to loosen the waist and stretch the back muscles. The stretches end with the class standing first on one leg and then on the other with the other leg raised bent at the knee and held up with one arm. Most people wobble as they try to balance in this position.

At 8.36 the capoeira instruction begins.

This is a typical start to every class Claudio delivers to adults. Claudio is clearly the fittest and most agile person in the room, although over 40. One experienced student Ikki occasionally steps away and does back stretches on his own in a corner. His back problems are well-known to Claudio who lets him warm himself up in his own way without comment.

While the individual prevention strategies that students use in private are not visible to the ethnographers, the shared injury prevention of the class warm up is public. Individual experienced students may comment that the general class stretches do not have enough emphasis on their particular “at risk” body area, and they will add more on their back, or shoulders or whatever. Others, like Ikki, will even step out of the lines if they know not to do a particular exercise.

Another preventative strategy is partaking in yoga and pilates, which were popular in themselves, as well as purposely used to prevent and control injuries.

I do yoga and I go to the gym (Female, 12 years, 3rd Belt)

Another woman, with 9 years of capoeira experience, on the 4th Belt, does “yoga regularly”, reports herself doing “more stretches” than the brief warm up before class, and wears wrist guards in class.

Another preventative strategy widely reported was avoiding capoeira moves that were beyond the student's competence or put strain on an area of the body that they perceive as vulnerable or weak. The informants we had were articulate about how they tried to avoid injuries. For example Rashka says:

I don't do things that I know my body is not ready for—well as far as I can. I don't do weird stuff for my shoulder. I basically don't try and learn something new until I've fixed my body. I have to really think about what I'm doing, or what I'm going to try and do. When people say “and try this move!” I don't, because I know the cost is too high. I always warm up myself before I come to lessons: a general warm up doesn't get me ready.

It never gets to the point where it never hurts at all, but I feel very lucky generally. If you strengthen and protect your body properly it can maybe decrease your chances of falling apart.

His prevention strategy is

about listening and being aware of your body and knowing what's going on and as long as you stay fully aware or try always to be listening and take the awareness of your body [seriously] then I think there is no reason why you should get injured.

Injuries happen “when you're not paying attention.” So his strategy is

go carefully, and listen to your body and place close attention to what things you're moving and how you're moving them.

The sixth strategy is capoeira specific, and is only reported retrospectively. Capoeira, when it is successful, with good music, strong loud singing, and rhythmic clapping, is characterized by good *axé*. *Axé* is a Yoruba word, used in the African-Brazilian religion *candomblé* to capture the power of the *orixás* (gods and goddesses). In capoeira it is used in a way equivalent to “The Force” in *Star Wars* to convey a positive force or energy, that enables people to play expressively, joyfully and exceed their “normal” skill level: in short it is the mystical energy that drives good capoeira as we have explained elsewhere (Stephens and Delamont, 2014; Delamont et al., 2017; Scott and Stephens, 2018). At festivals, and on special occasions, advanced students will report that the *axé* was so powerful it carried them through the problems they had with injuries. Abazi, scheduled to go up to his fifth belt at the 2018 winter festival arrived on the Saturday saying that he had been injured in his ribs on Friday, his feet were giving him trouble, and his recurrent back problems were bad. In his ceremonial games with seven visiting teachers he performed with no sign of any injuries and when asked by Delamont how he had managed to play said “The *axé* carried me through” (Stephens and Delamont, 2013; Delamont et al., 2017).

### Recovery Strategies

In this section we present and reflect on the data about the recovery strategies used by our informants. We thought that serious students who had experienced injuries might seek help from specialists in sport injuries, and consult therapists from alternative medicine, as well as using the British National Health Service (NHS). There is a considerable amount of self-treatment reported, with people saying that they know what to do from experience. Friends, and friends of friends who hold qualifications in physiotherapy, or sports medicine, or sports massage, or alternative therapies such as acupuncture are sources of advice, diagnosis and treatment. Family doctors are seen as ignorant about capoeira, and as lacking any facilities that can be used to deal with capoeira injuries, except referral to NHS physiotherapists or orthopedic surgeons. Referrals are not highly regarded because they take too long, and so NHS physiotherapists or other interventions are a last resort. If people can afford it, they use orthodox and alternative therapies privately: that is they pay for as many sessions as they can manage. The capoeira clubs we have researched include a good many people who have medical or other therapeutic qualifications and jobs, and so the injured capoeirista has access to a range of expertise and advice from their friends who are also capoeira enthusiasts.

## Discussion and Conclusions

Drawing on the injury narratives collected from capoeira enthusiasts we have found that Frank's typology of illness narratives can be usefully deployed to separate out different types of account. In their reflexive work on interviewing rugby players with spinal cord injuries Sparkes and Smith (2012) reject a standard realist account of the narratives they collected, and argue for a “reflexive” positive of *indwelling*: *feeling* their way into and out of the data through their known sensual corporeality (54–55). They argue that, when working with data such as our injury narratives researchers need empathy and sensual corporeality, and must never compromise the alterity of the informants. That was hard for Sparkes and Smith (2012) because of the severity of the spinal injuries and type of narrative the victims produced. In our case, the restitution stories that our informants gave Duarte and Lloyd were, once categorized, life enhancing for the social scientist and the capoeiristas.

We have drawn two related sociological conclusions from this investigation which are informed by our methodological conclusion. Because of the caveats we have about our informants, who were self-selected volunteers and did not include people who were temporarily or permanently absent from capoeira due to injury or other circumstances, we did not collect any chaos stories, and only two quest accounts. We suspect that other sampling methods among past and current students of diasporic capoeira could generate chaos and quest narratives. However, taking the restitution narratives as a corpus of data on capoeira we are confident that two useful conclusions can be drawn.

First, taking the position of Atkinson (1997) and Silverman (2017) seriously, these narratives are valuable when analyzed. They reveal features of the shared culture of serious capoeira students in the UK that they have acquired during their enculturations into its habitus. Like many features of any acquired habitus those apparent from these data are largely tacit or indeterminate, and become “taken for granted”. Indeed it was not until those narratives were analyzed that the authors realized discourse in and around injuries revealed features of the acquired habitus. We had “known” about them but not recognized their importance for a sociological understanding.

Experienced students have developed a range of explicit strategies to prevent injuries, and acquired explicit knowledge of a range of therapies, orthodox and alternative, are likely to ameliorate any injuries they do get. Being an advanced capoeirista is dependent on having these two bodies of knowledge. At the tacit or indeterminate level they have learnt to “listen to” their bodies. This is a shared skill, evidence of an acquired habitus of embodiment. As soon as this appeared during our analysis all the authors had a jolt of recognition, but one that we had not made explicit for ourselves or our readers before. This analysis of the restitution narratives, the useful ideal type proposed by Frank, when the narratives were not simply celebrated but worked on as sociological data, have exposed to us an important feature of the embodied habitus of diasporic capoeira previously unrecognized and unreported.

## Data Availability Statement

The datasets generated for this article are not readily available. There are two types of data: interviewed transcripts and ethnographic fieldnotes. The field notes only exist in handwritten form and are archived at SD's house. The interview transcripts contain highly confidential medical details, and would be very hard to anonymise. They are held in hard copy by SD. The original recordings were on TD and IL's phones and have been deleted once transcribed. Informants were not asked to give consent to any archiving or storing of their interviews: they were promised that only TD, IL, SD, and NS would see them. The data were collected in 2014 and 2016 and at least half of the respondents are no longer in contact with any of the four authors. Requests to access the datasets should be directed to neil.stephens@brunel.ac.uk.

## Ethics Statement

The studies involving human participants were reviewed and approved by Cardiff University School of Social Science Research Ethics Committee. The patients/participants provided their written informed consent to participate in this study. Written informed consent was obtained from the individual(s) for the publication of any potentially identifiable images or data included in this article.

## Author Contributions

The interview data were collected by IL and TD. The interviews were transcribed by IL and TD. Interviews conducted in Portuguese by TD were translated by TD. The data were analyzed by SD and NS, who also wrote the paper, drawing on discussions with TD. All authors agreed to the submission of the paper and contributed to the research reported in the paper.

## Conflict of Interest

The authors declare that the research was conducted in the absence of any commercial or financial relationships that could be construed as a potential conflict of interest.
